# Physiological and Behavioural Responses of Cattle to High and Low Space, Feed and Water Allowances During Long Distance Transport in the South of Chile

**DOI:** 10.3390/ani9050229

**Published:** 2019-05-10

**Authors:** Grisel Navarro, Viviana Bravo, Carmen Gallo, Clive J. C. Phillips

**Affiliations:** 1 Centre of Animal Welfare and Ethics, School of Veterinary Science, University of Queensland, Gatton, Queensland 4343, Australia; c.phillips@uq.edu.au; 2 Instituto de Ciencia Animal, Facultad de Ciencias Veterinarias, OIE Collaborating Centre for Animal Welfare and Livestock Production Systems—Chile, Universidad Austral de Chile, Valdivia 5090000, Chile; viviana.bravo.o@gmail.com (V.B.); cgallo@uach.cl (C.G.)

**Keywords:** transport, space allowance, cattle

## Abstract

**Simple Summary:**

Regarding the topography of Chile, livestock have to travel for long distances (1240 km), including road and sea transport. During this journey food and water deprivation, overstocking and handling issues have been reported as common problems. The lack of adequate conditions onboard can produce adverse consequences for the welfare of livestock in transit. In the current study the physiological and behavioural implications of two different space allowances and the lack of food and water during the long-distance transport has been studied. The current space provided in Chile to the cattle onboard (1 m^2^/500 kg) appears to be detrimental to their welfare. Lack of room to allow postural adjustment in combination with limited access to food and water resulted in poor nutrition and dehydration. Behavioural responses to the low space were also affected. Cattle increased the time spend eating and ruminating when 30% extra space and more feed was provided. We conclude that, under the current Chilean standards for the long-distance transport, the welfare of the livestock is adversely affected. This research can provide valuable information for the reform of welfare standards for the transport of livestock in Chile.

**Abstract:**

Long distance transport of livestock from Patagonia to central Chile involves both road and sea transport and has a potential impact on the welfare of animals. Fifty *Bos taurus* cattle of approximate age six months were exposed to a journey of four days, with both the sea and road components undertaken in a truck (roll-off roll-on system) with two pens of different dimensions. Thirty-two and eighteen cattle were randomly allocated to two treatment groups: Low and High welfare standards, which were provided 0.66 m^2^/head and 0.86 m^2^/head, respectively, and a fixed amount of feed and water daily to each pen, 1.25 kg hay/head and 3.1 L water/head in the Low welfare treatment and 2.22 kg/head and 5.6 L/head in the High welfare treatment, respectively. Low welfare animals had increased plasma total protein and albumin, which is suggested to be due to limited water availability, and also haptoglobin, suggesting inflammatory responses. Cattle in the High welfare treatment spent more time eating and ruminating than those in the Low space allowance, but they had increased cortisol at the end of the journey, perhaps reflecting increased fighting with more space. Cattle welfare in both treatments was adversely affected by the limited feed and water supplies, with increased beta-hydroxybutyrate at the end of the voyage; total protein was increased in just the low welfare standard group where low space allowance and less food and water was provided. Creatine phosphokinase also increased after the journey, compared with before, indicating bruising. Limiting feed and water availability to cattle in the low welfare treatment resulted in physiological evidence of undernutrition and low hydration status, but it also reduced the stress response, probably because there was less fighting.

## 1. Introduction

**Figure 1:**
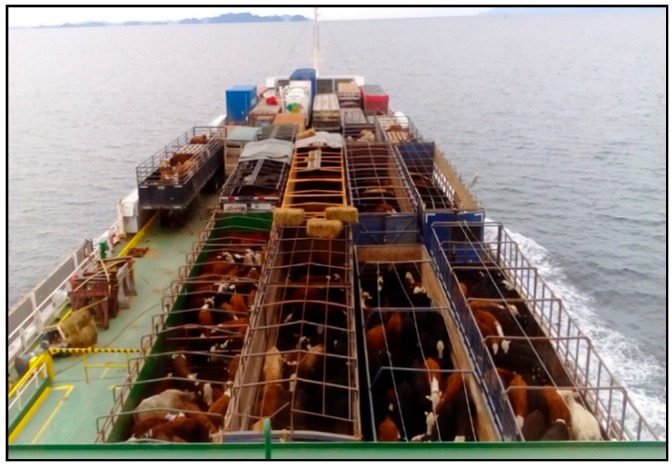
Transport of cattle from the Chilean Patagonia to the central-southern region of Chile using a roll-on/roll-off system for trucks.

Long distance transport of livestock in Chile is a common practice both between farms and from farms to a slaughterhouse [1]. Animals are transported long distances from the production centres to the main consumption areas, both because there are not enough slaughterhouses accredited for export at a regional level and because prices are higher at consumption centres [2,3]. Furthermore, the southernmost Patagonian region of Chile is characterized by a lack of good pasture for fattening [4], and therefore animals need to be sent into fattening farms. This long-distance transport necessarily includes transport by road and ferry due to the absence of roads in the south of the country. The ferry transport uses a roll-on/roll-off system, in which the livestock remain on trucks that are loaded and parked on the ferry (Figure 1).

This is one of the longest routine journeys for any livestock in South America [2], and it has been reported that the animals are frequently transported with limited water and food for 1700 km and for durations of more than 72 h [1]. In addition, overstocking and handling issues during loading, transport and unloading have been reported in several South American countries [2]. The current Chilean standard allows a minimum space of just 1 m^2^ for cattle of 500 kg bodyweight (500 kg/m^2^) [5], compared with other countries with higher standards for animal welfare during ship transport such as Australia, where the space required for cattle with similar bodyweight is 1.725 m^2^ on ship voyages of less than 10 days [6].

The lack of adequate conditions during long distance transport can reduce the welfare of livestock in transit, which can be assessed by behavioural and physiological measures [7]. One of the most common risks is a shortage of food and/or water. The former is typified by increased plasma free fatty acid (FFA), beta-hydroxybutyrate (BHB), urea and decreased glucose, and the latter by increased osmolality, total protein, albumin, and packed cell volume (PCV) [7,8,9]. PCV, total proteins and plasma albumin are convenient and simple measures of dehydration [10]. 

Space allowance is an important moderator of the animals’ ability to access food and water [11]. Space available for animals onboard ships has not been adequately studied for cattle subjected to long distance transport. The limited studies that have been conducted suggest that during transport cattle can lose considerable amounts of bodyweight, and that freedom to perform normal behaviours may be constrained by low space allowances. For instance, cattle transported from Ireland to Spain using a roll on/roll off system, with a space allowance of 0.9 m^2^/animal, lost 7.6 % (heifers) and 7.0% (bulls) of their initial live weight during the sea crossing from Ireland to France [12]. Similar weight loss occurs during road transport: weight losses of 10.5% to 11.9% have been reported in steers after 24 h of road transport at a space allowance of 500 kg/m^2^ [13]. In a separate study Friesian steers transported by road at a high stocking density (1.03–1.08 m^2^/head) for 24 h had inhibited movement, with fewer changes in position, elevated cortisol and glucose, and difficulty rising, compared with medium and low densities respectively (1.19–1.24 m^2^/head; 1.33–1.41 m^2^/head) [14]. The space provided to an animal on board is crucial to perform essential behaviours [15], principally the maintenance of normal posture, postural adjustments and stability maintenance, without risk of injury or physiological stress [16]. Future studies should include a sufficiently large range of space allowances, as has been used in stocking density experiments with dairy cows in barns, which usually vary density by approximately 50% [17]. The only other work in the area of sea space allowances, which used sheep, reported few differences and compared only a 10% difference (+ or −) from Australian standard allowances [18].

Food and water intake during transport may also be influenced by space allowance during transport, as well as feed type, familiarity between the animals and their motivation to eat and drink. Competition for the resources will be greater if the food and water is restricted than if it is available ad libitum [11]. Black [19] described inanition and dehydration as the main causes of death during 24 days of sea transport at the relatively low space allowance of 0.3 m^2^/sheep. Smaller animals were excluded from the feeding area by more dominant ones, particularly in the latter part of the voyage. Often feed is provided per pen without careful consideration of the number of animals.

The objective of this current study was to determine the physiological and behavioural responses to two space allowances and levels of feed and water provision in pens of two different sizes with two different groups of cattle. Cattle were exposed to business as usual conditions in long distance transport by ship and truck from Patagonia to central Chile. The experiment was designed to test animal responses to the sea journey under commercial conditions, in order to determine the most stressful components. 

## 2. Materials and Methods

The study was conducted in the Patagonian region in the far south of Chile. Ethical approval was obtained from both universities involved, Austral de Chile and Queensland; approval numbers 254/2016 and AE1481, respectively. The study complied with sea transport standards for Chile [20].

### 2.1. Animals and Housing

Fifty weaned cattle, *Bos taurus* Hereford x Criollo dual-purpose cattle, of approximate 6 months of age and with a body weight of 171.9 ± 6.18 kg (mean ±SEM) were selected for the experiment. The cattle had been grazing on pasture dominated by *Festuca gracillima*, with water available from natural streams. The day before the truck loading, animals were moved 2 km from the pasture to a holding pen, where they remained for 12 h without feed or water. 

### 2.2. Experimental Protocol

The 50 cattle were exposed to commercial transport, including by road and sea over 4 days in total, travelling from Puerto Natales, in the Chilean Patagonia, to Osorno (1240 km north). The first and the last parts of the journey included road transport by truck, initially travelling from a farm 50 km away from Puerto Natales (1 h). The middle part of the journey involved ferry transport from the Puerto Natales to Puerto Montt, a distance of 1142 km (92 h). The final part of the journey, by truck was from Puerto Montt to Osorno, a distance of 101 km (2 h).

**Figure 2:**
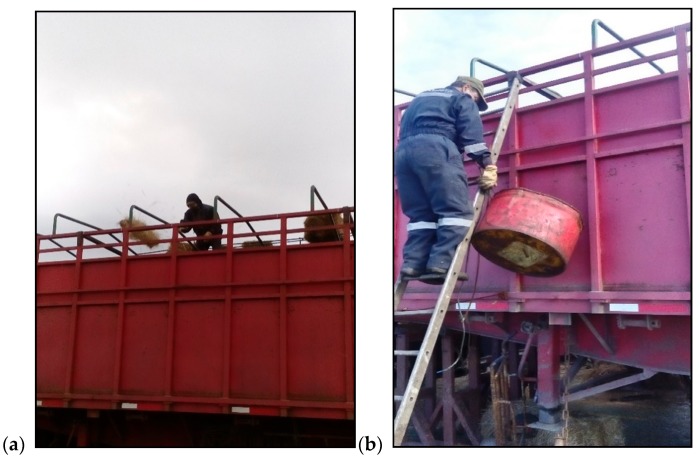
(**a**) Providing a bale of hay; (**b**) half of a barrel for watering a pen of cattle during sea transport.

At the start of the journey all cattle were loaded onto an open deck lorry, with metal flooring, sawdust bedding and natural ventilation, subdivided into two compartments, one for each treatment group, to which animals were randomly allocated. The treatments were based on space allowance and feed and water provision: 1) Low welfare (8.45 × 2.50 m^2^, providing 0.66 m^2^/head for 32 animals) and 2) High welfare (6.05 × 2.50 m^2^, providing 0.86 m^2^/head for 18 animals). The Low welfare allowance treatment was marginally greater than the Australian standard for the export or livestock (ASEL) [6] and was approximately twice that of the current cattle transport standard in Chile (500 kg/m^2^ or 0.34 m^2^/animal of the weight in our study, 171.9 kg) [5]. Animals in the High welfare treatment had 30% more space. During the road section of the journey (30 km from the farm to the ferry, a 1-h trip), the cattle did not receive any feed or water. During the ferry section a single bale (20 kg) of grass hay was thrown twice daily at 09:30 and 18:00 h into the middle of each pen, providing on average 1.25 and 2.22 kg/d to cattle in the Low and High welfare treatments, respectively (Figure 2a). Water was provided in a half fuel barrel, at a rate of 100 L per pen daily, 3.1 L/head and 5.6 L/head on average in the Low and High welfare treatments, respectively (Figure 2b).

### 2.3. Haematological Measurements

Ten animals per treatment were randomly selected for haematological measurements, identified by a colour mark on their back and the number in their plastic ear tags. The first sample was taken immediately before loading at the farm of origin, and the second when they arrived at the farm of destination. Blood samples were taken by venipuncture of the coccygeal vein into 9 mL heparinised tubes (Vacuette^®^tubes, Greiner Bio-One International, Kremsmünster, Austria) using a vacutainer 21 G needle (BD Vacutainer Eclipse, Franklin Lakes, NJ USA) and placed on ice for storage prior to analysis for cortisol, leukocytes, packed cell volume (PCV), betahydroxybutyrate (BHB), creatine-phosphokinase (CK), total protein (TP), globulin (G) and haptoglobin (Hp) concentrations. Samples were analysed using techniques previously reported to assess the welfare of cattle during transport [21]. Plasma cortisol concentrations were measured by radioimmunoassay (CLIA); plasma glucose by the enzymatic colorimetric Glucose oxidase-phenol amino phenazone (GOD-PAP) test; BHB values by an enzymatic technique based on 3-hydroxybutyratedehydrogenase enzyme, plasma CK by the International Federation of Clinical Chemistry (IFCC) and European Committee for Clinical Laboratory Standards (ECCLS) kinetic method, and serum haptoglobin using a commercial kit Tridelta PHASE Haptoglobin Assay (Tridelta Development Ltd., County Kildar, Ireland).

### 2.4. Behaviour Measurements

Cattle behaviours were video recorded during days 1–3 of the sea journey. Nine video cameras (Kobi CCD Video Camera, Model K-32HCVF, Ashmore, QLD, Brisbane, Australia) were positioned around the truck at appropriate locations to capture each treatment group’s behaviour. Behaviours were coded for a random but identifiable 6 animals/pen with the aid of the coding software CowLog (Cowlog 3.0.2, University of Helsinki, Helsinki, Finland), using a continuous sampling technique of 5 min observation per hour, the whole journey was assessed during the daylight time. To facilitate animal identification a paint mark was positioned on the back of each recorded animal during the loading procedure. The behaviours recorded, and their definition are in Table 1, selected from relevant behaviours in our previous work with simulated ship transport [22,23].

### 2.5. Statistical Analysis

The statistical package Minitab version 17 was used, with all values presented as means. A general linear model was used to analyse haematological parameters, with the following factors: animal as a random variable, and welfare (Low and High), sampling time (preloading Vs after unloading), and the interaction between these two factors as fixed factors. For behaviour the general linear model included animal, welfare, nested within animal, and day. The analyses were checked for normal distribution of residuals using the Anderson-Darling test. In both analyses post hoc Tukey’s and Fisher’s tests were used to identify significant differences between individual means, the latter where Tukey’s test did not discriminate between the means. For every statistical procedure, alpha was set to be less than 0.05 to be considered significant.

## 3. Results

### 3.1. Haematological Parameters

Over both measurements, before and after transport, animals in the Low welfare treatment increased albumin and total protein and had increased haptoglobin, compared with those in High welfare (Table 2). There were no significant treatment differences in PCV, neutrophils, cortisol, BHB, glucose, CK or globulin. With respect to the sampling time (Table 3), taking cattle in both treatments together, they had increased neutrophils, glucose, albumin and total protein before loading compared to after unloading at the destiny farm. After unloading, the cattle displayed increased BHB and CK compared to before loading.

There were interactions between sampling time and welfare treatment (Table 4). The cattle in the High welfare treatment had a greater cortisol concentration after arriving at the destination farm (7.26 mg/dL) compared with prior to the transportation event (5.62 mg/dL), however, there was no difference in the cattle in the Low welfare treatment. Regarding blood proteins, albumin was reduced after unloading in the High welfare treatment but in the Low welfare treatment they were high both before and after the journey. Globulin was reduced at the end of the journey in the High welfare treatment but increased in the Low treatment. In total, blood proteins were lowest after unloading in the High treatment, but were increased after unloading, compared with before the journey, in the Low treatment.

### 3.2. Behavioural Parameters

Cattle in treatment High spent more time eating and standing ruminating and less time standing not ruminating (Table 5), compared with animals with Low space allowance. They also spent more time lying down ruminating. With respect to the stages of the journey, on the second day the time that animals were standing eating and stepping increased, compared with the first and the last day of the journey (Table 6). There were no significant differences when the rest behaviours described in the ethogram were analysed.

## 4. Discussion

The long-distance transport and the space/feed/water treatments provided had an impact on the welfare of the cattle transported in a combined journey including land and sea in the south of Chile, as evidenced by effects on both physiological and behavioural measurements. We accept the confounding of space, feed and water provision factors in this study, but stress that this was on a commercial shipment, where there was little opportunity to set factors exactly as we would like, as usually occurs on commercial shipments. Nevertheless, the journey per se, as opposed to welfare level of provision, produced an increase in beta-hydroxy butyrate and a decrease in glucose concentration after unloading, probably associated with the lack of food during the journey or a disruption in the normal pattern of feeding. From the feed provision of 1.25 kg/head/d and 2.22 kg/head/d in the Low and High treatments, it can be calculated that cattle in the Low welfare treatment only received about 45% of their maintenance requirement, and those in the High welfare treatment 79%, assuming that standard maintenance allowances for 172 kg cattle are 24 MJ/d, or 2.4 kg DM (approximately 2.8 kg freshweight)/head/d of hay with 9 MJ/kg DM [24]. Increases in BHB after transport in cattle have been reported previously. Early [25] found a greater BHB after a transportation of bulls for 24 h by road. Also, Knowles [26] found increases in BHB after 18 h and 24 h of food deprivation in cattle transported by road. They associated this with the mobilization of body reserves in response to increasing inanition. In addition, Werner [4] reported that there was an increase in BHB values in calves after 68 h of road travel, which they suggested was in response to the food and water deprivation during the journey. Even though in the present study the animals were fed with a bale of fodder twice a day in each pen, it was apparently insufficient to satisfy their metabolic demands. This finding is supported by the decrease in glucose concentration at the end of the journey. The reduction of glucose is attributable to its utilization as a source of energy [23].

The increase in the values of the enzyme creatine phosphokinase after unloading indicates that there were physical demands and fatigue during the trip. The increase in CK after long distance transport has been reported by several authors [3,4,8,25], all suggesting that long-distance transport represents a physical challenge for the animals that causes muscle damage, likely related to bruising. Additionally, the increase in the cortisol levels after unloading in the high space allowance group suggests that the handling process for this group was more stressful for these cattle, conceivably because they had more room to interact during the journey, including aggressive interactions. It could be also explained by the sea movements being tolerated differently in the two treatments. The capability to maintain the balance when more space was available may have represented a big challenge for the animals and resulted in stress. However, recent research [22,23] indicates that the close proximity of other livestock during simulated ship motion may make movement more stressful, because the movement of those animals is unpredictable.

This, however, was not supported by increased CK in cattle in the High welfare treatment. Werner et al. [4] suggested that steers from the south of Chile living in extensive conditions with little contact with humans could have a major fear response to the handling procedures. Furthermore, they pointed out that the stress responses were exacerbated by the movement of cattle over a distance of 4–6 km from their farm to a waiting pen, and also by the weaning process before the transport.

The significant increase of total protein in the blood of cattle in the Low welfare treatment after unloading indicates dehydration. The decrease in the concentration of total proteins and albumin in the blood of cattle in the High welfare treatment after unloading could be explained by the fact that these cattle had easier access to water and less competition for this resource. Nevertheless, water provision was not enough to satisfy all the animals’ requirements, which is likely to be in the region of 15 L/d [27]. The fact that the Low and High treatments supplied one fifth and one third of requirements, respectively, is likely to be an underestimate of the stress caused to some animals, because distribution was probably not equal to all cattle in the group. Dehydration in the cattle in the Low welfare treatment may have been the reason for the increase in globulin by the end of the journey, whereas in the High welfare treatment globulin may have decreased because of the stress of the journey [28]. Even if stress was greater in the Low treatment compared to the High treatment, this may have been over-ridden by the haemoconcentration as a result of dehydration. These effects were not supported by PCV differences between treatments, probably because this reflects differences in red blood cells, whose output may have been maintained during water deprivation, whereas protein concentration may have declined due to a deprivation of both feed and water, leading to a greater likelihood of detection differences between treatments.

Haptoglobin is an acute phase protein that is often used as a stress, inflammation, trauma and infection marker, and in this study, it was within the reference range [29]. It functions to scavenge and bind metabolites, in particular haemoglobin released from erythrocytes during cellulolysis, thereby inhibiting oxidative activity and sequestering iron to retard bacterial growth [30]. The notable increase in the Low welfare treatment suggests that these cattle had increased stress, as a result of inflammatory responses. Four to six hours of tethered transport are enough to increase haptoglobin concentrations in cattle [31], as is transport of feedlot calves over 1400 km in two days [32].

The cattle in the High space allowance spent more time eating and ruminating, both standing and lying, and less time standing not ruminating. This is not surprising given their greater feed allocation. Rumination is a crucial indicator of wellbeing when animals are confined for several days. Provision of greater allowances of feed would be expected to further increase this welfare parameter.

## 5. Conclusions

Long distance transport from Patagonia in the south of Chile to central Chile adversely affected the welfare of the cattle. There was evidence of physiological stress, inadequate water and feed provision, with increased space, feed and water apparently reducing dehydration. Stress biomarkers showed contradictory results, especially between cortisol and creatine kinase. Behaviour indicators suggested greater comfort at high space, feed and water allowances.

## Figures and Tables

**Table 1:** Ethogram describing the behaviours recorded for cattle (*n* = 12) in High and Low space allowances during two road and one ship journey.

Behaviours	Definition
Standing	Standing with the four legs over the floor
Lying down	Resting with the whole body on the floor
Walking	Moves forward or backward with the four legs (more than one body length)
Stepping	Balancing against the sea motion by spreading their feet
Turned around	The front part of cow’s body turned around to the back
Displacement	Moves to where another is lying who then moves away
Displaced	Moves away when another comes over to its location
Self-grooming	Licking a part of their body
Allogrooming	Licking a part of another animal’s body
Rumination whilst lying and standing	Chewing movements whilst ruminating
Head above partner	The head of the animal is over the body of another conspecific
Butting	Head to head encounter
Mounting	An animal climbs on the back of another

**Table 2:** Mean concentrations of blood variables of cattle at two different welfare levels, Low and High. Low 0.66 m^2^/head and High 0.86 m^2^/head. (The standard error of the mean (SEM) was used on the table)

Variable	Welfare		SEM	*F*-Value	*p*-Value
	Low	High			
Packed cell volume (%)	34.0	33.4	0.43	0.96	0.33
Neutrophils (mill/μL)	0.57	0.69	0.06	2.01	0.17
Cortisol (mg/dL)	6.55	6.44	0.35	0.05	0.82
Beta-hydroxy butyrate (mmol/L)	0.41	0.37	0.05	0.27	0.60
Glucose (mmol/L)	4.80	4.60	0.22	0.39	0.53
Creatine phosphokinase (U/L)	905	705	84.2	2.26	0.14
Total proteins (g/L)	77.0	73.3	0.78	11.04	0.002
Albumin (g/L)	47.6	45.0	0.39	22.42	<0.001
Globulin (g/L)	29.3	28.3	0.57	1.63	0.21
Haptoglobin (g/L)	0.50	0.27	0.07	4.62	0.04

**Table 3:** Mean concentrations of blood variables of cattle before and after a journey by truck on the road and by sea, averaged across two different space allowances, Low 0.66 m^2^/head and High 0.86 m^2^/head.

Variable	Sampling Time		SEM	*F*-Value	*p*-Value
	Before Loading	After Unloading			
Packed cell volume (%)	34.2	33.1	0.43	2.83	0.10
Neutrophils (mill/μL)	0.98	0.27	0.06	62.8	<0.001
Cortisol (mg/dL)	6.28	6.72	0.35	0.81	0.37
Beta-hydroxy butyrate (mmol/L)	0.29	0.50	0.05	8.37	0.007
Glucose (mmol/L)	5.25	4.15	0.22	12.1	0.002
Creatine phosphokinase (U/L)	662	949	94.1	4.66	0.04
Total protein (g/L)	76.1	74.1	0.78	3.47	0.07
Albumin (g/L)	47.3	45.2	0.40	13.97	0.001
Globulin (g/L)	28.8	28.8	0.57	0.00	0.97
Haptoglobin (g/L)	0.36	0.40	0.07	0.17	0.68

**Table 4:** Means of blood variables of cattle subjected to long distance transport and interactions between loading/unloading sampling and welfare level. Low 0.66 m^2^/head and High 0.86 m^2^/head.

Welfare	Low		High		SEM	*F*-Value	*p*-Value
Before/After Journey	Before	After	Before	After			
Packed cell volume (%)	34.3	33.6	34.1	32.7	0.60	0.33	0.56
Neutrophils (mill/μL)	0.92	0.21	1.05	0.34	0.09	0.00	0.98
Cortisol (mg/dL)	6.93 ^ab^	6.17 ^ab^	5.62 ^b^	7.26 ^a^	0.49	5.98	0.02
Beta-hydroxy butyrate (mmol/L)	0.25	0.58	0.34	0.41	0.07	3.31	0.08
Glucose(mmol/L)	5.43	4.17	5.07	4.13	0.31	0.26	0.61
Creatine phosphokinase (U/L)	750	1060	572	838	133	0.03	0.87
Total proteins (g/L)	75.3 ^b^	78.6 ^a^	77.0 ^ab^	69.6 ^c^	1.10	22.8	<0.001
Albumin (g/L)	47.7 ^a^	47.5 ^a^	47.0 ^a^	43.0 ^b^	0.56	11.83	0.002
Globulin (g/L)	27.6 ^b^	31.0 ^a^	30.0 ^a^	26.6 ^b^	0.80	3.27	<0.001
Haptoglobin (g/L)	0.40	0.57	0.31	0.22	0.10	0.86	0.21

Different letters within a row indicate significant differences (*p* < 0.05) between low and high space allowances.

**Table 5:** Effects of Low and High welfare provision on behaviour responses of cattle during the transport by sea. Low 0.66 m^2^/head and High 0.86 m^2^/head.

Behaviour (%/day)	Welfare		SEM	*F*-Value	*p*-Value
	Low	High			
Standing					
Ruminating	3.70	13.2	0.0249	7.31	0.02
Not ruminating	84.3	54.6	0.0452	21.7	<0.001
Eating	2.30	11.8	0.0168	16.0	0.001
Lying down					
Ruminating	0.17	5.09	0.0166	4.37	0.05
Not ruminating	0.06	0.10	0.0272	1.32	0.269
Others					
Stepping (step/h)	152	139	18.2	0.23	0.64
Walking	0.01	0.01	0.0059	0.31	0.59
Self grooming	0.0006	0.0004	0.0003	0.19	0.67

**Table 6:** Effects of the day of the journey in behavioural responses of cattle subjected to long distance transport by sea.

Behaviour	Day			SEM	*F*-Value	*p*-Value
	1	2	3			
Standing eating (%/day)	4.31 ^b^	11.6 ^a^	5.15 ^b^	0.02	3.87	0.044
Stepping (*n*/day)	118 ^b^	211 ^a^	108 ^b^	22.3	6.47	0.009

Different letters within a row indicate significant differences (*p* < 0.05) between days.
